# Base-Metal Heterogeneous Catalysts for Upgrading Plastics to Light Olefins

**DOI:** 10.34133/research.0731

**Published:** 2025-06-10

**Authors:** Zhen Yu, Yafei Fan, Fawei Lin

**Affiliations:** ^1^ Department of Mechanical Engineering, City University of Hong Kong, Kowloon 999077, Hong Kong.; ^2^ Key Laboratory for Colloid and Interface Science of Ministry of Education, School of Chemistry and Chemical Engineering, Shandong University, Jinan 250100, China.; ^3^ School of Environmental Science and Engineering, Tianjin University/Tianjin Key Lab of Biomass/Wastes Utilization, Tianjin 300072, China.

## Abstract

Developing low-cost plastic recycling technologies is crucial for ecological sustainability and the circular economy. The recent publication in *Science* by Conk et al. introduces an innovative method employing base-metal catalysts, specifically WO_3_/SiO_2_ and Na/γ-Al_2_O_3_, to efficiently convert polyethylene, polypropylene, or their mixtures into valuable products, representing a marked advancement in the field of base-metal catalysis and plastic recycling.

Plastics have become the third largest production material around the world with a total output of 400 million tons per year. Accordingly, the nonbiodegradable property of plastics brings about 6,300 million ton accumulation of waste, inducing severe environmental issues, which would cause CO_2_ emission and microplastic pollution during direct incineration and landfill [1,2]. Recycling and upgrading of plastics are the most suitable approaches to promoting environmental protection and sustainability rather than simple landfill and incineration [3–5]. Breaking down plastics into their original monomers is theoretically an ideal recycling strategy. Thermal pyrolysis and gasification operating at a higher temperature, ca. ≥500 °C, can effectively cleave polyolefins with production of multitype gases, oils, and char, and catalyzed hydrogenolysis at a certain pressure has also been reported to attain depolymerization. For instance, Martín et al. [6] proposed a tandem hydrogenolysis/aromatization catalyzed by Pt/γ-Al_2_O_3_ (Pt loading of 1.5 wt %) to convert polyethylene (PE) into long-chain alkyl aromatics and alkyl cycloalkanes. However, most existing methods suffer from low selectivity, necessitating further separation and upgrading, substantial production of greenhouse gas, and reliance on noble metal catalysts [7]. In response, Conk et al. [8] reported a more sustainable pathway by leveraging base-metal heterogeneous catalysts to convert plastics into valuable products in a recent *Science* article.

They meticulously combined an olefin metathesis catalyst WO_3_/SiO_2_ and an olefin isomerization catalyst Na/γ-Al_2_O_3_ that are both used industrially to generate propylene and isobutylene mixture by tandem-heterogeneous catalysis of catalytic cracking and isomerizing ethenolysis (CIE) under 15 bar of ethylene at 320 °C for 90 min. Converting a polyolefin into light olefins like propylene (global market: 169 million tons and US$22.0 billion in 2023) and isobutylene (valued at US$25.9 billion in 2024, key precursor of fuels and polymers) has high economic benefits and simultaneously reduces greenhouse gas emissions compared to incineration. Chain cleavage without skeletal isomerization and concomitant isomerization of olefins over these basic catalysts selectively generated propylene from PE and mixture of propylene and isobutylene from polypropylene (PP) (Figure A). Interestingly, Na/γ-Al_2_O_3_ and WO_3_/SiO_2_ enhanced the above 2 steps to a larger extent, respectively, for PE and PP (Figure B). Particularly, this approach exhibited a distinct advantage in the high selectivity of targeted olefins with no ethane or propane that causes difficult separation, thereby contributing to being less energy intensive for postpurification.

**Figure.**
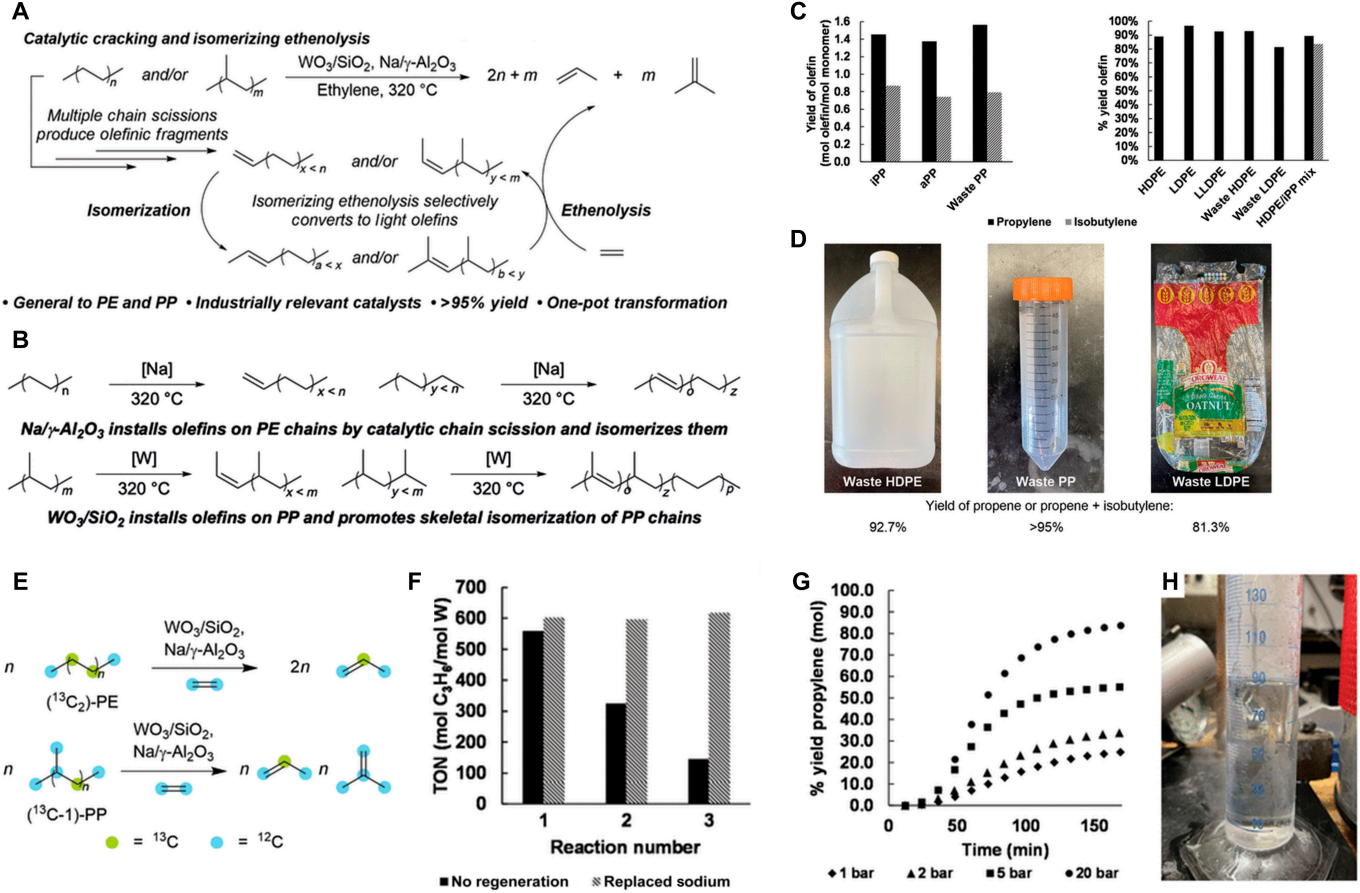
Upgrade and transformation of plastics by base-metal catalysts [8]. (A) Schematic diagram of catalytic cracking and isomerization to produce light olefins from polyethylene (PE) and polypropylene (PP). (B) The role of Na/γ-Al_2_O_3_ and WO_3_/SiO_2_ catalysts. (C) Propene and isobutylene yields of plastic polymers from different sources. (D) Photographs of sources of waste subjected to cracking and isomerizing ethenolysis (CIE). (E) Isotopic labeling experiments with unlabeled PE and PP. (F) Investigation of catalyst recyclability. (G) Investigation of the effect of ethylene pressure on the yield of high-density polyethylene (HDPE) CIE in a semibatch reactor. (H) Large-scale production of liquefied propylene and butene mixtures. iPP, isotactic polypropylene; aPP, atactic polypropylene; LDPE, low-density polyethylene; LLDPE, linear low-density polyethylene; TON, turnover number.

Na/γ-Al_2_O_3_ and WO_3_/SiO_2_ composite catalysts achieve effective conversion of PP from various sources, so that the ratio of propylene to isobutene is about 2:1; high-density PE is mainly converted to propylene and the yield is as high as 87% (Figure C). More importantly, for waste plastics, propylene (C_3_H_6_) and isobutylene (C_4_H_8_) can still be obtained efficiently through catalytic CIE (Figure D). Investigating the impact of various feedstocks, including other types of plastic waste, could also broaden the scope of this catalytic system. Such versatility in catalyst performance suggests a robust approach to utilizing mixed plastic waste, addressing one of the major challenges in the field of plastic recycling. This initial comparison demonstrates the selectivity of the catalysts toward producing alkenes from polyolefins, showcasing the potential for efficient conversion without requiring extensive pretreatment or separation processes.

The isotope labeling experiments confirmed that propylene was mainly derived from polyolefin rather than ethylene (Figure E). The catalytic system could retain 50% activity after each cycle with a more than 10 times higher total turnover number than previous molecular metathesis catalysts. Nevertheless, further addition of a fresh Na/γ-Al_2_O_3_ could completely demonstrate good cyclic stability. Finally, the feasibility of CIE in a semibatch reactor was further validated by optimizing the operation temperature and residence time, as well as enhancing the mass transport by stirring the reactants (Figure F to H). The great advantages of low cost, good stability, and high conversion rate indicate great potential for industrial applications, especially in the context of circular economy initiatives.

Additionally, the study provides a clear road map for developing similar catalytic processes that can tackle other types of plastic waste. The feasibility of using the base-metal catalyst for high-yield conversion has been proven. By further exploring the catalysts, like high-activity single-atom catalysts and catalytic processes for upcycling, that can be used for polyolefin conversion, researchers may discover new and even more efficient ways to dispose of plastic waste [9–11]. The integration of catalytic conversion technologies with existing waste management methods could substantially enhance the overall efficiency of plastic recycling efforts. This approach aims to transform solid waste plastics in the environment into high-value multifunctional catalysts through low-energy-consumption methods, enabling their effective utilization and achieving sustainable waste management [12,13]. While the results are promising, several avenues for future research could augment the applicability of this technology. Conducting comprehensive life cycle assessments will be essential to quantify the environmental benefits of the proposed method relative to those of existing plastic recycling techniques. To enhance catalyst stability, designing core–shell architectures or doping WO_3_/SiO_2_ with transition metals (e.g., Fe and Co) could mitigate sintering and carbon deposition. Coupling in situ regeneration protocols (e.g., oxidative treatments) with reactor engineering may further extend catalyst lifespan. For mixed plastics, integrating artificial-intelligence-driven near-infrared sorting systems with pretreatment modules (e.g., density-based separation) can ensure feedstock uniformity, while developing multi-zone catalytic reactors tailored to varied polymer degradation kinetics may enable simultaneous processing. In terms of halogenated plastics, such as polyvinyl chloride, dichlorination pretreatment is essential to couple with the CIE process; otherwise, integrating halogen-tolerant catalysts (e.g., Fe-doped WO_3_) to address Cl^−^ poisoning should be incorporated. Synergizing advanced sorting with adaptive catalytic systems promises scalable, efficient plastic upcycling aligned with circular economy goals.

In conclusion, the research by Conk et al. marks an important step forward in catalytic recycling technologies for polyolefins. This work provides a practical method for the degradation of polyolefin while reducing greenhouse gas emissions. The tandem catalytic process operates at 320 °C (vs. ≥500 °C for pyrolysis), reducing energy demand by ~40%. More than 95% conversion of carbon was achieved by the tandem catalytic process, contributing to substantial reduction in CO_2_ emissions. Furthermore, the reduced energy consumption also contributes to fewer indirect CO_2_ emissions. As the world grapples with the environmental challenges posed by plastic waste, studies like this are vital in paving the way for a more sustainable future. As the study demonstrates, the implementation of such technologies could substantially contribute to reducing plastic waste and promoting sustainability within the chemical industry. This work not only contributes to the academic literature but also provides practical implications that could influence policy and industrial practices in recycling and waste management.
